# The impact of high versus standard enteral protein provision on functional recovery following intensive care admission (PRECISE trial): study protocol for a randomized controlled, quadruple blinded, multicenter, parallel group trial in mechanically ventilated patients

**DOI:** 10.1186/s13063-023-07380-3

**Published:** 2023-06-19

**Authors:** Rob J. J. van Gassel, Julia L. M. Bels, Katrien Tartaglia, Bas C. T. van Bussel, Sander M. J. van Kuijk, Adam M. Deane, Zudin Puthucheary, Peter J. M. Weijs, Lilian Vloet, Bert Beishuizen, Ashley De Bie Dekker, Vincent Fraipont, Stoffel Lamote, Didier Ledoux, Clarissa Scheeren, Elisabeth De Waele, Arthur R. H. van Zanten, Dieter Mesotten, Marcel C. G. van de Poll

**Affiliations:** 1 Department of Intensive Care Medicine, Maastricht University Medical Center, Maastricht, the Netherlands; 2 NUTRIM School for Nutrition and Translational Research in Metabolism, Maastricht University, Maastricht, the Netherlands; 3 Department of Surgery, Maastricht University Medical Center, Maastricht, The Netherlands; 4 Clinical Trial Unit, Ziekenhuis Oost-Limburg, Genk, Belgium; 5 Cardiovascular Research Institute Maastricht (CARIM), Maastricht University, Maastricht, the Netherlands; 6 Department of Clinical Epidemiology and Medical Technology Assessment, Maastricht University Medical Center, Maastricht, the Netherlands; 7 Department of Critical Care, Melbourne Medical School, University of Melbourne, Parkville, Australia; 8 William Harvey Research Institute, Barts and The London School of Medicine & Dentistry, Queen Mary University of London, London, UK; 9 Adult Critical Care Unit, Royal London Hospital, London, UK; 10 Department of Nutrition and Dietetics, Faculty of Sports and Nutrition, Amsterdam University of Applied Sciences, Amsterdam, the Netherlands; 11 Department of Nutrition and Dietetics, Amsterdam University Medical Center, Vrije Universiteit, Amsterdam, the Netherlands; 12 Research Department of Emergency and Critical Care, HAN University of Applied Science, School of Health Studies, Nijmegen, the Netherlands; 13 IQ Healthcare, Radboud Institute for Health Sciences, Radboud University Medical Center, Nijmegen, the Netherlands; 14 Department of Intensive Care Medicine, Medisch Spectrum Twente, Enschede, the Netherlands; 15 Department of Intensive Care, Catharina Hospital, Eindhoven, the Netherlands; 16 Service of Intensive Care Medicine, Citadelle Hospital, Liège, Belgium; 17 Department of Intensive Care Medicine, AZ Groeninge, Kortrijk, Belgium; 18 Sensation & Perception Research Group, GIGA Consciousness, University of Liège, Liège, Belgium; 19 Intensive Care Units, University Hospital of Liège, Liège, Belgium; 20 Department of Intensive Care Medicine, Zuyderland Medisch Centrum, Heerlen/Sittard, the Netherlands; 21 Departement of Nutrition, Universitair Ziekenhuis Brussel, Jette, Belgium; 22 Department of Intensive Care, Gelderse Vallei Hospital, Ede, the Netherlands; 23 Department of Intensive Care Medicine, Ziekenhuis Oost-Limburg, Genk, Belgium; 24 Faculty of Medicine and Life Sciences, UHasselt, Diepenbeek, Belgium

**Keywords:** Enteral nutrition, Nutrition therapy, Dietary protein, Critical illness, Functional outcomes

## Abstract

**Background:**

Critically ill patients are subject to severe skeletal muscle wasting during intensive care unit (ICU) stay, resulting in impaired short- and long-term functional outcomes and health-related quality of life. Increased protein provision may improve functional outcomes in ICU patients by attenuating skeletal muscle breakdown. Supporting evidence is limited however and results in great variety in recommended protein targets.

**Methods:**

The PRECISe trial is an investigator-initiated, bi-national, multi-center, quadruple-blinded randomized controlled trial with a parallel group design. In 935 patients, we will compare provision of isocaloric enteral nutrition with either a standard or high protein content, providing 1.3 or 2.0 g of protein/kg/day, respectively, when fed on target. All unplanned ICU admissions with initiation of invasive mechanical ventilation within 24 h of admission and an expected stay on ventilator support of at least 3 days are eligible. The study is designed to assess the effect of the intervention on functional recovery at 1, 3, and 6 months following ICU admission, including health-related quality of life, measures of muscle strength, physical function, and mental health. The primary endpoint of the trial is health-related quality of life as measured by the Euro-QoL-5D-5-level questionnaire Health Utility Score. Overall between-group differences will be assessed over the three time points using linear mixed-effects models.

**Discussion:**

The PRECISe trial will evaluate the effect of protein on functional recovery including both patient-centered and muscle-related outcomes.

**Trial registration:**

ClinicalTrials.gov Identifier: NCT04633421. Registered on November 18, 2020. First patient in (FPI) on November 19, 2020. Expected last patient last visit (LPLV) in October 2023.

**Supplementary Information:**

The online version contains supplementary material available at 10.1186/s13063-023-07380-3.

## Administrative information

**Table:** 

Title {1}	The impact of high versus standard enteral protein provision on functional recovery following intensive care admission: a randomized controlled, multicenter, parallel group trial in mechanically ventilated, critically ill patients; PRotEin provision in Critical IllneSs—PRECISe trial
Trial registration {2a and 2b}	ClinicalTrials.gov Identifier: NCT04633421, registered November 18^th^, 2020
Protocol version {3}	Clinical Study Protocol Version 4.0, December 15^th^, 2022 (the Netherlands) Clinical Study Protocol Version 3.0, December 15^th^, 2022 (Belgium)
Funding {4}	Funding for this trial was provided by the BeNeFIT program of the The Netherlands Organisation for Health Research and Development (ZonMW) and the Belgian Health Care Knowledge Center (KCE) Blinded enteral nutrition for the trial was provided in kind by Nutricia Research, Utrecht, the Netherlands
Author details {5a}	Rob J.J. van Gassel M.D. ^1,2,3^, Julia L.M. Bels M.D. ^1,2^, Katrien Tartaglia ^4^, Bas C.T. van Bussel M.D. Ph.D. ^1,5^, Sander van Kuijk Ph.D.^6^, Adam Deane M.D. Ph.D. ^7^, Zudin Puthucheary M.D. Ph.D. ^8,9^, Peter Weijs M.D. Ph.D. ^10,11^, Lilian Vloet Ph.D. ^12,13^, Bert Beishuizen M.D. Ph.D. ^14^, Ashley de Bie Dekker M.D. Ph.D. ^15^, Vincent Fraipont M.D. Ph.D. ^16^, Stoffel Lamote M.D. Ph.D. ^17^, Didier Ledoux M.D. Ph.D. ^18,19^, Clarissa Scheeren M.D. ^20^, Elisabeth De Waele M.D. Ph.D. ^21^, Arthur van Zanten M.D. Ph.D. ^22^, Dieter Mesotten* M.D. Ph.D. ^23,24*^ Marcel C.G. van de Poll* M.D. Ph.D. ^1,2,3*^ **Affiliations** ^1^ Department of Intensive Care Medicine, Maastricht University Medical Center, Maastricht, the Netherlands ^2^ NUTRIM School for Nutrition and Translational Research in Metabolism, Maastricht University, Maastricht, the Netherlands ^3^ Department of Surgery, Maastricht University Medical Center, Maastricht, The Netherlands ^4^ Clinical Trial Unit, Ziekenhuis Oost-Limburg, Genk, Belgium ^5^ Cardiovascular Research Institute Maastricht (CARIM), Maastricht University, Maastricht, the Netherlands ^6^ Department of Clinical Epidemiology and Medical Technology Assessment, Maastricht University Medical Center, Maastricht, the Netherlands ^7^ Intensive Care Unit, Royal Adelaide Hospital, Adelaide, SA, Australia ^8^ William Harvey Research Institute, Barts and The London School of Medicine & Dentistry, Queen Mary University of London, London, UK ^9^ Adult Critical Care Unit, Royal London Hospital, London, UK ^10^ Department of Nutrition and Dietetics, Faculty of Sports and Nutrition, Amsterdam University of Applied Sciences, Amsterdam, the Netherlands ^11^ Department of Nutrition and Dietetics, Amsterdam University Medical Center, Vrije Universiteit, Amsterdam, the Netherlands ^12^ Research Department of Emergency and Critical Care, HAN University of Applied Science, School of Health Studies, Nijmegen, the Netherlands ^13^ IQ healthcare, Radboud Institute for Health Sciences, Radboud university medical center, Nijmegen, the Netherlands ^14^ Department of Intensive Care Medicine, Medisch Spectrum Twente, Enschede, the Netherlands ^15^ Department of Intensive Care, Catharina Hospital, Eindhoven, The Netherlands ^16^ Service of Intensive Care Medicine, Citadelle Hospital, Liège, Belgium ^17^ Department of Intensive Care Medicine, AZ Groeninge, Kortrijk, Belgium ^18^ Sensation & Perception Research Group, GIGA Consciousness, University of Liège, Liège, Belgium ^19^ Intensive Care Units, University Hospital of Liège, Liège, Belgium ^20^ Department of Intensive Care Medicine, Zuyderland Medisch Centrum, Heerlen/Sittard, the Netherlands ^22^ Department of Intensive Care, Gelderse Vallei Hospital, Ede, the Netherlands ^21^ Departement of Nutrition, Universitair Ziekenhuis Brussel, Jette, Belgium ^23^ Department of Intensive Care Medicine, Ziekenhuis Oost-Limburg, Genk, Belgium ^24^ UHasselt, Faculty of Medicine and Life Sciences, Diepenbeek, Belgium *DM and MvdP share co-senior authorship
Name and contact information for the trial sponsor {5b}	Marcel C.G. van de Poll, MD, PhD Department of Intensive Care Medicine Maastricht University Medical Center + , The Netherlands Email: marcel.vande.poll@mumc.nl
Role of sponsor {5c}	Sponsor: Together with the Belgium coordinating center (Ziekenhuis Oost-Limburg, Genk): Concept, design, and coordination of the study. Responsibility for the integrity of the data and analysis and writing and publication of the report

## Introduction

### Background and rationale {6a}

Critical illness is characterized by a protein catabolic state, resulting in severe skeletal muscle wasting during ICU stay [1]. Loss of muscle mass and function adversely affects both short- and long-term outcomes, with persistent physical disability impacting long-term quality of life during ICU recovery [2–4]. Interventions aimed at attenuating the catabolic state during critical illness could help diminish the rate of muscle loss and enhance post-ICU functional recovery and quality of life [5]. Preservation of muscle mass and function in critically ill patients are key objectives for both scientific and patient representative’s research agendas [6, 7].

Dietary protein is an important anabolic stimulus and paramount in preserving muscle mass in healthy subjects [8]. Unfortunately, clinical trials on nutritional interventions in ICU patients so far have infrequently assessed functional and muscle-related endpoints, despite the physiological rationale [9–11]. Several retrospective studies suggest a beneficial role for increased dietary protein, separate from calorie delivery [12, 13] and a systematic review suggested benefit of greater protein regarding mortality, though not enough trial data was available to draw any firm conclusions [14]. In the absence of high-level prospective evidence, current European and American nutrition guidelines vary in their recommendations for protein targets: 1.3 g/kg/day and 1.2–2.0 g/kg/day, respectively [15, 16]. Therefore, prospective trials assessing the impact of increased dietary protein provision on patient recovery are placed at the top of the ICU nutritional research agenda [17]. Recently, a large randomized trial achieved 1.6 g/kg/day of protein compared to 0.9 g/kg/day in the control group and found no difference in mortality or ICU length of stay, yet no functional outcomes were assessed [18]. As other large nutrition trials also failed to demonstrate an effect on mortality [19–23], the importance of assessment of functional outcomes instead of mortality as a primary endpoint is further underlined. Finally, post-ICU recovery trajectories may evolve and differ over time, which cannot be captured by survival analyses only [24]. Hence, assessment of functional and quality of life outcomes at different time points over a time horizon of at least 90 days, and preferably up to 1 year, after ICU admission, may be the preferred follow-up of ICU outcome in clinical practice and trials with nutritional interventions [24–26].

In the current study, we hypothesize that provision of enteral nutrition with increased protein content during critical illness is able to enhance functional recovery following ICU admission. The study will assess recovery over time up until 6 months using functional, muscle-related, and patient-centered outcomes [27].

### Objectives {7}

The PRECISe study will randomly allocate mechanically ventilated patients admitted to the ICU between enteral nutrition containing a high or standard amount of protein. The high protein nutrition will amount to 8 g/100 kcal (or 2.0 g/kg/day) when reaching full nutritional targets, compared to 5 g/100 kcal (or 1.3 g/kg/day) for the standard protein nutrition. Functional recovery of participants will be assessed at 1, 3, and 6 months from ICU admission. The primary objective is to compare differences between the two study arms over time in health-related quality of life (HRQL) assessed by the Euro-QoL-5D-5-level (EQ-5D-5L) questionnaire. Secondary objectives include the effect on overall survival, physical performance, muscle and nerve function, and mental health. These outcomes are derived from the core outcome set (COS) of the Outcomes After Critical Illness and Surgery (OACIS) Group, registered in the COMET initiative database [15].

### Trial design {8}

The PRECISe study is a bi-national, quadruple-blinded, multi-center randomized controlled trial (RCT) with a parallel-group design and a 1:1 allocation ratio assessing the superiority of high versus standard protein provision in mechanically ventilated ICU patients. A visual overview of the trial design is provided in Supplemental Figure S1.

## Methods: participants, interventions, and outcomes

### Study setting [9]

The study will be conducted in critically ill patients admitted to the ICUs of 10 hospitals across Belgium and the Netherlands. These hospitals contain a balanced mix of both general and tertiary academic referral centers. The full list is provided in the supplemental documents.

### Eligibility criteria [10]

Adult patients with an unplanned admission to the ICU and initiation of invasive mechanical within 24 h of admission were screened for enrolment using the inclusion and exclusion criteria as specified below.

### Inclusion criteria

- Adult (18 years or above) patient admitted to ICU
- Unplanned ICU admission
- Invasive, mechanical ventilation initiated < 24 h following ICU admission
- Expected ICU stay on ventilator support of 3 days or more

### Exclusion criteria

- Contraindication to enteral nutrition
- Moribund or withholding of treatment
- Kidney failure AND a “no dialysis”-code on ICU admission
- Hepatic encephalopathy (West Haven grades 3–4)
- Body-mass index < 18 kg/m.^2^

### Who will take informed consent? {26a}

Due to the nature of the study population and selection criteria, patients who are eligible for study participation will not have the capacity to provide informed consent. As such, initial informed consent will be obtained from the patient’s proxy (i.e., a legal representative). Consistent with local and national laws, acting as the patient’s proxy is identified, using a hierarchical model including (in descending order): (1) a court-appointed legal representative, (2) a patient’s authorized legal representative, (3) a husband/wife, (4) a registered partner or other life companion, (5) parents, (6) children of the patient that are of age, or (7) brothers or sisters that are of age and can reasonably be contacted. Patients eligible for study participation will be identified by treating physicians, who will inform the local study team. A member of the treatment team will ask the patient’s proxy permission to be approached by a member of the study team to initiate the informed consent procedure.

Patient recruitment and initiation of study nutrition can occur before written informed consent is obtained using a deferred consent procedure (The Netherlands) or after written informed consent by a proxy is obtained (Belgium) according to national law, respectively. In both cases, the patient’s proxy will be informed as soon as possible and asked to provide written informed consent. If patients regain capacity to provide informed consent to continue participation, they will be asked to provide written informed consent themselves. At any time, the patient or their legal representative can refuse or withdraw consent for the study without providing a reason and without impacting the treatment provided. The study protocol, consent forms, and relevant documentation were approved by the Medical Ethics committee of Maastricht University (METC azM/MUMC + , METC20-039) and Leading Ethical Committee of the Universitair Ziekenhuis Brussel (2020/223).

### Additional consent provisions for collection and use of participant data and biological specimens {26b}

For any mechanistic and exploratory studies outside the aims and scope of the primary study protocol, additional institutional review board approval and informed consent from the patient or their proxy will be obtained.

## Interventions

### Explanation for the choice of comparators {6b}

For both study arms, a simple pragmatic nutritional protocol will be used, adhering to the current guidelines of the European Society for Enteral and Parenteral Nutrition, which recommend the initiation of enteral nutrition within 48 h after ICU admission and restriction of energy provision to < 80% of the estimated energy expenditure during the first 3 days of ICU admission, followed by a full coverage of energy expenditure from day 4 onwards [15]. Thus, enteral nutrition (EN) is to be initiated within 48 h of ICU admission, with energy targets set at 25 kcal/kg/day to be reached on day 4 of admission. As both study feeds are isocaloric, the caloric target will translate in a daily volume target of 20 ml/kg/day for both groups. To avoid overfeeding in the acute phase, enteral nutrition will be commenced at 25% of calculated target and increased by a further 25% per day until 100% of energy targets are reached on day 4. To avoid overfeeding in overweight and obese patients, the weight used to calculate volume targets will be actual body weight for those with BMI $\le$ 27 kg/m^2^ and ideal body weight for patients with a BMI > 27 kg/m^2^. The formula used to calculate ideal body weight is $27 \times {height}^{2}$. The study nutrition will be continued for the duration of ICU stay if enteral nutrition is required or until a maximum of 90 days. Patients that have commenced enteral nutrition remain eligible provided they meet all of the inclusion criteria and none of the exclusion criteria. Data on type and total volume of administered nutrition will be collected.

### Intervention description {11a}

Applying the standardized nutrition protocol detailed above, patients will be randomized between two study arms representing two enteral, isocaloric nutrition formulas that differ in their protein content:

*The standard protein arm* uses an enteral formula containing a standard amount of protein (Nutrison Protein Plus (Nutricia, Zoetermeer, the Netherlands), 5 g protein/100 kcal, 1.25 kcal/ml). From day 4 onwards and when fed to the target rate of 20 ml/kg/day, patients in the standard protein arm will receive 25 kcal/kg/day and 1.26 g protein/kg/day. This amount of protein is consistent with the recommendations in the ESPEN and at lower end of the range recommended in the ASPEN guidelines [15, 28].

*The high protein arm* uses an enteral formula containing a greater concentration of protein (Nutrison Protein Intense (Nutricia), 8 g protein/100 kcal, 1.25 kcal/ml). From day 4 onwards and when fed to the target rate of 20 ml/kg/day, patients in the high protein arm will receive 25 kcal/kg/day and 2.0 g protein/kg/day. This amount of protein is consistent with the high end of the range recommended in the ASPEN guideline [28].

### Criteria for discontinuing or modifying allocated interventions {11b}

The allocated study intervention will be discontinued in case of:

- Death, or ICU discharge, or after a maximum of 90 days
- On demand of the treating physician (i.e., sufficient oral intake or contra-indication to continue EN)
- On demand of the patient or their proxy

In case the patient is readmitted to the ICU within 48 h of discharge of index ICU admission, the intervention and daily data collection will be resumed. In case the patient is readmitted after 48 h of discharge of index ICU admission, the readmission will be scored as such in the eCRF, but the intervention and daily data collection will not be resumed. In case the patient is transferred to another non-study site ICU, the intervention and daily data collection are stopped. In all cases, patients are followed until 180 days after index ICU admission for collection of primary and secondary endpoints. Under no circumstances will patients be deliberately switched to a study arm other than the arm the patient was assigned to.

### Strategies to improve adherence to interventions {11c}

Frequent feeding interruptions in the ICU often cause a mismatch between the amount of nutrition prescribed and received by the patient. To improve nutritional adequacy when providing full enteral nutrition from day 4 onwards, the volume provided will be subtracted from the daily volume target of 20 ml/kg/day. If the provided volume falls short of the volume prescribed, the deficit will be added to the volume target for the next treatment day (catch-up feeding). In addition, gastric residual volumes will not be routinely measured, as this practice lowers nutritional adequacy without preventing occurrence of ventilator-associated pneumonia [29, 30]. All study sites were extensively trained in the intervention protocol before start of the trial and prepared their study site to facilitate the execution of the nutrition protocol.

### Relevant concomitant care permitted or prohibited during the trial {11d}

During the first 7 days of ICU stay parenteral nutrition will be prohibited [15, 20]. If after 7 days, a patient’s daily nutritional targets cannot be met via enteral nutrition due to severe gastro-intestinal dysfunction, use of supplemental parenteral nutrition is permitted, but enteral nutrition remains the preferred route of delivery, and all efforts will be made to switch to full enteral nutrition and wean from parenteral nutrition as soon as possible. This will include the use of prokinetics and placement of a post-pyloric feeding tube according to local practice. Enteral protein supplements are prohibited. The amount of administered non-nutritional calories will be collected, but no adjustments to the nutritional targets will be made based on this.

### Provisions for post-trial care {30}

All participants will be monitored independently from the study according to clinical standards. Follow-up of participants within the trial will occur up until 6 months following ICU admission. There are no additional provisions for post-trial care. The sponsor has insurance to cover damage to participants through injury or death directly caused by study participation. Travel costs for participants to attend follow-up visits will be reimbursed.

### Outcomes {12}

Primary and secondary outcomes will be collected during ICU stay and follow-up at approximately 30, 90, and 180 days following ICU admission. The outcome measures are based on the extended core outcome set for acute respiratory failure survivors [27]. Overall between-group differences will be assessed over the three time points using linear mixed-effects regression analysis (see the “ Statistical analysis plan” section).

### Primary outcome

The primary outcome is health-related quality of life. This will be assessed by the overall difference in EQ-5D-5L health utility score between intervention and control group over three time-points (30, 90, and 180 days after ICU admission), adjusted for baseline EQ-5D-5L. The EQ-5D-5L consists of a 5-item questionnaire evaluating the following domains: mobility, self-care, usual activities, pain/discomfort, and anxiety/depression. The responses to the 5-item questionnaire can be converted into a 5-digit number, which reflects a unique health state for that participant. Next, the health state is weighted using the country specific value sets for the Netherlands and Belgium, respectively, resulting in the EQ-5D-5L health utility score [31, 32]. The health utility score ranges from − 0.532 to 1.0, with a score of 0 indicating death, a score below 0 a state worse than death, a higher score indicating better health, and a score of 1 indicating perfect health.

The EQ-5D-5L is a standardized measure of health status already used in several multicenter clinical trials, including previous nutrition trials, and has become widely recognized as a valid and well-noted instrument for measuring health status by patient and health care decision makers [33–37]. The EQ-5D-5L is valid for both patient and proxies and has been translated in different languages (https://euroqol.org/eq-5d-instruments).

### Secondary outcomes

The following secondary outcomes will be collected at 30, 90, and 180 days after ICU admission:

- Overall survival
- Health-related quality of life assessed by the Short Form 36 (SF-36)
- Anxiety and depression, assessed by the Hospital Anxiety and Depression Scale (HADS)
- Pain intensity assessed by the EQ-5D-5L pain question
- Self-reported health assessed by the EQ-5D-5L visual analogue scale (EQ-VAS)
- Post-traumatic stress assessed by the Impact of Event Scale Revised (IES-R)
- Physical function assessed by 6-min walk distance
- Muscle and nerve function assessed by Medical Research Council (MRC)-sum score
- Muscle and nerve function assessed by handgrip strength

In landmark studies on functional outcome in prolonged critically ill patients, the validated 36-item SF-36 questionnaire has been used as indication for health-related quality of life [4]. Therefore, the SF-36 questionnaire will be collected, and both an overall score derived from the eight health domains assessed, as well as the physical component score (PCS) and mental component score (MCS), will be calculated. Furthermore, we will collect the EQ-5D-5L visual analogue scale (EQ-VAS) to assess self-rated health on a scale of 0–100 and report the EQ-5D-5L pain question separately as is recommended by the core outcome set [27]. The Hospital Anxiety and Depression Scale (HADS) is a validated questionnaire to evaluate a patient’s level of anxiety and depression, for which both the overall HADS score and sub scores for anxiety and depressive symptoms will be calculated and reported according to established methods [38]. Lastly, the Impact of Event Scale Revised (IES-R), a 22-item questionnaire that evaluates a patient’s subjective distress level caused by a traumatic event, and the Rockwood Clinical Frailty Scale will be collected. The Rockwood Clinical Frailty Scale is a simple nine-point scale that clinicians can use to assess the frailty level of a patient. It incorporates the evaluation of mobility, energy level, physical, and functional activity [39]. Similarly to the EQ-5D-5L, the Rockwood Clinical Frailty Scale will be collected both at baseline and during follow-up.

Aside from the hypothesized general improvement of health-related quality of life due to provision of higher enteral protein (and the attenuation of catabolic processes), it can be hypothesized that the driving factor behind this improvement is preservation of muscle strength during critical illness [40]. Measures to assess physical and muscle function will be assessed to investigate whether increased enteral protein provision will result in increased physical and muscle strength*.* The 6-min walk distance evaluates functional exercise capacity by measuring the self-paced distance walked in 6 min. The test will be performed according to a standardized protocol, including assessment of pre- and post-test heart rate and saturation [41]. The absolute distance walked and the percentage of predicted will be collected and use of any aids (e.g., oxygen therapy, walking aid) will be registered [42]. The Medical Research Council (MRC)-sum score appraises bilateral strength for six muscle groups (shoulder abduction, elbow flexion, wrist extension, hip flexion, knee extension, and ankle dorsal flexion). Each group will be scored on a five-point scale ranging from 0 (no visible contraction) to 5 (normal strength) per group and amount to a maximum score of 60 [43]. Lastly, handgrip strength, a validated, objective, and robust measure of peripheral skeletal muscle function, will be assessed using a hand dynamometer [44]. The maximum value out of three attempts per side will be collected as absolute value and as percentage of predicted [45]. Because muscle weakness is a key determinant of long-term functional outcomes and quality of life, improvements in physical functioning should also translate into improved quality of life (SF-36, especially the physical component score), pain level (assessed by the pain question in the EQ-5D-5L questionnaire), and mental health status (assessed by HADS, IES-R and EQ-VAS) [2, 4].

Although prospective data have never shown any effect of nutrition on mortality, there is retrospective data and point estimates from systematic reviews suggesting that more protein might improve survival [13, 14].

### Tertiary outcomes

- Duration of mechanical ventilation (i.e., number of days on invasive mechanical ventilation)
- Duration of index ICU stay (i.e., number of days in ICU)
- Duration of index hospital stay (i.e., number of days in hospital)
- Hospital mortality
- Sixty-day mortality
- Time-to-discharge-alive (i.e., days until live hospital discharge)
- Nutritional adequacy (i.e., ratio between total amount of calories and grams of protein actually received by patients and prescribed during treatment period)
- Administration of prokinetics (i.e., number of patients who received a prokinetic and number of days receiving a prokinetic drug)
- Incidence of gastrointestinal intolerance/symptoms (i.e., number of patients that experienced, at any time during index ICU stay, vomiting, ischemia, diarrhea, abdominal distention, gastric paresis, or bleeding/ulcer)
- Incidence of ICU readmissions (i.e., number of patients readmitted to the ICU during index hospital stay and number of readmissions per patient)
- Incidence of ICU acquired infections (i.e., number of patients who contracted an ICU-acquired infection)
- Incidence of acute kidney injury (i.e., number of patients with Acute Kidney Injury (AKI), defined as a serum creatinine level higher than 2 times baseline level)
- Incidence and duration of renal replacement therapy (i.e., number of patients who received renal replacement therapy and days on it)
- Incidence of hepatic dysfunction (i.e., number of patients with hepatic dysfunction, defined as a total bilirubin level > 3 mg/dL)
- Maximum and mean Sequential Organ Failure Assessment (SOFA) score
- Difference in mobilization treatment (i.e., number of days and degree of daily mobilization (passive/active, in-bed cycling, etc.))
- Difference in frailty assessed by Rockwood Clinical Frailty Scale, adjusted for baseline
- Domain data EQ-5D-5L (i.e., scores of subdomains of EQ-5D-5L at each 30, 90, and 180 days)
- Destination of hospital discharge (i.e., home, rehabilitation center, care facility, etc.)
- Length of stay at rehabilitation facility (i.e., number of days at rehabilitation center)
- Time to return to work (i.e., number of days between ICU admission and return to work)
- Health economic analysis (total health care costs)

### Participant timeline {13}

The study period starts with a screening and enrolment phase at ICU admission. After randomization and treatment allocation, the treatment phase starts and continues for the duration of ICU stay or a maximum of 90 days. During this phase, patients receive enteral nutrition according to the allocated treatment arm and study nutrition protocol. Follow-up occurs at approximately 30, 90, and 180 days after admission to the ICU. A detailed time schedule of trial procedures can be found in Table 1.

**Table 1:** Trial procedures

**PRECISe study**	**Screening and enrolment**	**Treatment phase**		**Follow-up phase** (during ICU stay or after ICU discharge)		
**Time point**	**On ICU admission**	**Daily during ICU stay** (max 90 days after ICU admission)	**Only on treatment day 1, 3, 5, 7, 9, 11, and 13**	**FU visit 1** (30 days ± 4 days after ICU admission)	**FU visit 2** (90 days ± 4 days after ICU admission)	**FU visit 3** (180 days ± 4 days after ICU admission)
**Enrolment**						
Eligibility screen, informed consent, randomization	X					
**Intervention**						
Initiate study feeding/re- adjust study feeding rate		X				
Daily volume of nutrition received		X				
**Data collection**						
Demographics, medical history, admission information, NRS-2002 ^a^	X					
Vital signs ^b^, laboratory findings ^c^, GCS score, fluid balance, SOFA score items	X		X			
Ventilation status		X				
Mobilization treatment ^d^		X				
Concomitant medication ^e^	X	X				
**Assessments**						
*Primary outcome* (EQ-5D-5L)	X			X	X	X
*Secondary outcomes* (SF-36, 6MWD, MRC-sum, HGS, HADS, IES-R)				X	X	X
Rockwood Clinical Frailty Index	X			X	X	X
ICU events		_X_^f^		X	X	X
Post-ICU discharge information ^g^				X	X	X

^a^Including age, sex, pre-admission weight and height, comorbidities according to the Charlson Comorbidity Index, APACHE IV admission diagnosis, and variables required for disease severity scores (APACHE, SAPS, SOFA)

^b^Highest and/or lowest values recorded during the 24-h treatment day

^c^Hemoglobin, hematocrit, white blood cell count, platelets, CRP, urea, creatinine, K^+^, PO_4_, magnesium, albumin, bilirubin, ALT, AST, GGT, ALP, pH, glucose, PaO_2_/FiO_2_ ratio. Only on admission: Na^+^, bicarbonate, lactate

^d^Mobilization received during treatment day: passive/active in bed, passive/active on bed bicycle, electrostimulation, out of bed

^e^Glucocorticoids, antibiotics, prokinetics, muscle relaxants, and medication/infusions with substantial non-nutritional calories (e.g., insulin, propofol, citrate)

^f^All-cause mortality, ICU readmissions, life threatening event caused by the nutrition (SAE), hepatic dysfunction (i.e., cholestasis and liver dysfunction), ICU acquired infections (including ventilator-associated pneumonia (VAP)), ECMO, acute kidney injury (including the use of renal replacement therapy), refeeding hypophosphatemia and gastrointestinal events (i.e., vomiting, ischemia, diarrhea, abdominal distention, gastric paresis, and/or bleeding/ulcer)

^g^Destination of discharge, length of stay at rehabilitation center, return to work (if applicable)

### Sample size {14}

Trial data of health-related quality of life scores repeated at multiple time points after ICU discharge are not readily available. For this reason, the power calculation was based on a published point-measurement of the EQ-5D-5L health utility score at 180 days following ICU admission [46]. Reported instrument-defined minimally important difference estimates for the EQ-5D-5L utility scores are between 0.037 and 0.069 for Canada, China, Spain, Japan, England, and Uruguay scoring algorithms [47, 48]. We chose a minimum difference of 0.06 points on the EQ-5D-5L health utility score as representing the minimum clinically important difference to be detected [47].

Based on these data, the sample size for the PRECISe trial was calculated as follows: with an estimated standard deviation (SD) of health utility scores of 0.3 at 180 days [46], considering a type I error rate *α* = 0.05 and a type II error rate *β* = 0.20 (yielding a statistical power of 80%), 392 participants per intervention group will be required to detect the minimum clinically important difference of 0.06 in EQ-5D-5L health utility score. In line with other critical care trials, the sample size has been adjusted upwards for an estimated 5% loss to follow-up for the primary endpoint [19]. After this adjustment, the final sample size for the PRECISe trial was set at 824 participants.

During the preplanned interim safety analysis, it became apparent that mortality was higher than anticipated, resulting in a standard deviation of the EQ-5D-5L HUS that was larger than expected (0.38 vs 0.30). Since this potentially could reduce the power of the study, the DSMB advised to review the statistical analysis plan to account for this and agreed on increasing sample size. By running a computer simulation of the primary outcome analysis using actual pooled data of the actual study population at that time (*n* = 709), it was calculated that with the observed standard deviation, a sample size of 935 patients would be required to retain 80% power to detect the minimally important difference of 0.06, while correcting for the actually observed loss-to-follow-up of 9.4%.

### Recruitment {15}

Local study teams have screened every unplanned admission to the ICU with initiation of invasive mechanical ventilation within 24 h of admission for eligibility. The screening procedure has been collected on a screening log. Study teams reported which patients were or were not included and, if not included, documented the reason why the patient was not included. The sponsor was alerted with each randomization and kept track of inclusion rates per site, in order to steer sites where recruitment rates drop or stay behind.

## Assignment of interventions: allocation

### Sequence generation {16a}

A computer algorithm was used to generate the random allocation sequence. Patients were randomized in a 1:1 ratio, using block randomization with random permuted block sizes varying between 4 and 6. Randomization of patients was stratified by center to account for systematic differences in routine practice between the participating centers.

### Concealment mechanism {16b}

Randomization was performed centrally through an interactive web response system (IWRS). Due to the combination of central randomization and varying block sizes, sites were not able to guess the treatment assignment based on the block size.

### Implementation {16c}

The enteral nutrition used for this study was blinded by the supplier of the enteral formulas (Nutricia, The Netherlands). The allocation sequence was generated by an independent member of the clinical trial unit in Maastricht managing the IWRS software (ALEA, ALEA Clinical, Abcoude, The Netherlands) in collaboration with an independent member of the enteral formula supplier.

Eligible patients were randomized via the IWRS (ALEA, ALEA Clinical, Abcoude, The Netherlands) by a trained member from the local study team. This generated a unique study randomization number and feeding label (A, B, C, or D), which corresponds with the labels placed on the enteral nutrition bottles. The generated feeding label corresponds to either a high or standard enteral protein formula depending on the patient’s allocation. Thus, while randomization occurred between two intervention groups (high or standard protein), each intervention group has two representing feeding labels, to prevent complete unblinding of the study in the event that unblinding of a label occurs.

## Assignment of interventions: blinding

### Who will be blinded {17a}

This is a quadruple-blinded study; meaning that participants, care providers, investigators, and outcome assessors are blinded to the assigned intervention. Furthermore, data analysts and the independent safety monitoring committee will be blinded. Nutritional intervention will be blinded and marked with a feeding label (A, B, C or D) per nutritional unit without anyone involved in trial execution, outcome assessment, or data analysis having any knowledge to which study arm each feeding label belongs. It has already been established that these feeds can be administered in a blinded fashion [36]. Before data analysis, the independent member managing the IWRS software (ALEA) will reveal which two separate codes belong together as 1 intervention group to the trial statistician for data analysis. Only after all data analyses will have been performed, the study group concealment will be lifted.

### Procedure for unblinding if needed {17b}

Although no serious adverse events (SAE) related to the study nutrition are expected, unblinding is possible under strict conditions. The study code should only be broken for valid medical or safety reasons, where it is necessary for the investigator or treating health care professional to know which treatment the patient is receiving before the participant can be treated. An emergency telephone number is available 24/7 to obtain unblinding information. It is not mandatory, but strongly encouraged, to contact the chief investigator before unblinding any patient’s treatment assignment. Unblinded data are to be kept strictly confidential until the time of unblinding of the trial and will not be accessible by anyone else involved in the trial with the following exceptions: (1) the product manager of the company responsible for the labeling and packaging of the nutritional product, (2) the Interactive Response Technologies system programmers who work on the randomization and drug management system, and (3) the data manager who prepares reports required for regulatory reporting. These individuals will not be involved in the day-to-day operation of the trial.

## Data collection and management

### Plans for assessment and collection of outcomes {18a}

An overview of trial procedures, including data collection and assessment of outcomes, is presented in Table 1. Data collection regarding patient characteristics, admission information, relevant concomitant medication, incidence of prespecified events of special interest (see Table 1), and clinical characteristics on admission and during ICU and hospital stay will be collected from patient records. The EQ-5D-5L will be completed on admission by a proxy to be able to assess baseline, pre-admission health-related quality of life. In addition, the Nutritional Risk Screening (NRS-2002) and Rockwood Clinical Frailty Scale will be completed on admission, using information provided by the proxy or medical records.

During the first 2 weeks of ICU stay, detailed information on vital signs, laboratory results, and items required for the SOFA score will be recorded every other day. The amount of nutrition, propofol, insulin, and mobilization treatment received by the patient is collected daily.

In addition to routine clinical data, specialized assessments will be performed at 30, 90, and 180 days after ICU admission. A window ranging from 4 days before to 4 days after the calculated follow-up date is defined, within which outcomes will be assessed. These outcomes include questionnaires (i.e., EQ-5D-5L, HADS, SF-36, IES-R) and physical tests (i.e., handgrip strength, 6-min walk distance, MRC-sum score). Physical tests will be performed by experienced, trained personnel based on a standardized manual of operations in all centers.

Finally, general information not detailed in the patient records will be assessed during follow-up. These include if and when patients returned to work, duration of stay at a rehabilitation center (if applicable) and the patient’s survival status. If deceased, the date of death will be collected as a secondary endpoint.

In the event a participant is not able to return to the hospital for assessment, when possible, a house visit or telephone call will be performed to collect all possible endpoints.

Local ancillary sub-studies collecting and storing plasma, urine, or fecal material as well as the collection of indirect calorimetry, bioelectrical impedance analysis (BIA), and muscle ultrasound data are nested within the overall trial. These will be reported separately and fall beyond the scope of this manuscript.

### Plans to promote participant retention and complete follow-up {18b}

Maximum effort will be made by the local study teams to have participants complete the study follow-up assessments. Travel expenses for participants will be fully covered. If participants are unable or unwilling to return to hospital, telephone calls or house visits will be made to collect as much outcome data as possible. Reasons for drop-out or not attending follow-up will be recorded.

In case of study withdrawal, all data collected up until the moment of withdrawal will be retained unless objections are made by the participant or their proxy. In that case, all data collected will be destroyed.

### Data management [19]

All study data will be recorded and stored in an electronic case report form (eCRF) created with the CASTOR© software (Castor, Amsterdam, The Netherlands). To protect the privacy of the participants, all collected data will be encoded, consisting of a code specific for the site of recruitment, the abbreviation of the study (PRECISe), and an incremental 3-digit number per center (starting from 001 in order of inclusion). CASTOR© complies with all applicable medical data privacy laws and regulations: GCP, 21 CFR Part 11, EU Annex 11, the European Data Protection Directive, ISO9001, and ISO27001/NEN7510. The data will be entered by trained staff who are registered on the training and delegation log. The PRECISe eCRF has been equipped with automatic checks that issue queries when impossible or unlikely values are entered. Furthermore, entered data is checked by a trained data manager on inconsistencies or impossible/unlikely values. When all data will have been checked and deemed correct, the data are to be locked by the data manager, after which the principal investigator of each study site signs off the records in the eCRF. Lastly, 10% of all entered data and 100% of data regarding serious adverse events is checked by the study monitor, who will perform source data verification. In case of significant errors, source data verification will be increased to 100% of all entered data for sites involved.

### Confidentiality {27}

Only the identification log and informed consent forms will contain patient names and will be stored locally in a secure location at each participating center only accessible for authorized members of the local study team. No personal identifying information will be stored in the eCRF.

### Plans for collection, laboratory evaluation, and storage of biological specimens for genetic or molecular analysis in this trial/future use {33}

No biological specimens will be collected or stored for the primary analysis of this trial. Local ancillary sub-studies collecting and storing plasma, urine, or fecal material are nested within the overall trial. These will be reported separately and fall beyond the scope of this manuscript.

## Statistical methods

### Statistical analysis plan

#### Statistical methods for primary and secondary outcomes {20a}

Here, we present the statistical analysis plan for the PRECISe trial with regard to the primary and secondary endpoints. The full details of the statistical methodology will be described in the statistical analysis plan. The full statistical analysis plan will be finalized before database lock and submitted as a supplement with the eventual study paper. Additionally, a Bayesian analysis of the trial will be performed, of which the methodology will also be described in the statistical analysis plan.

#### General rules of the statistical analyses

**Fig. 1:**
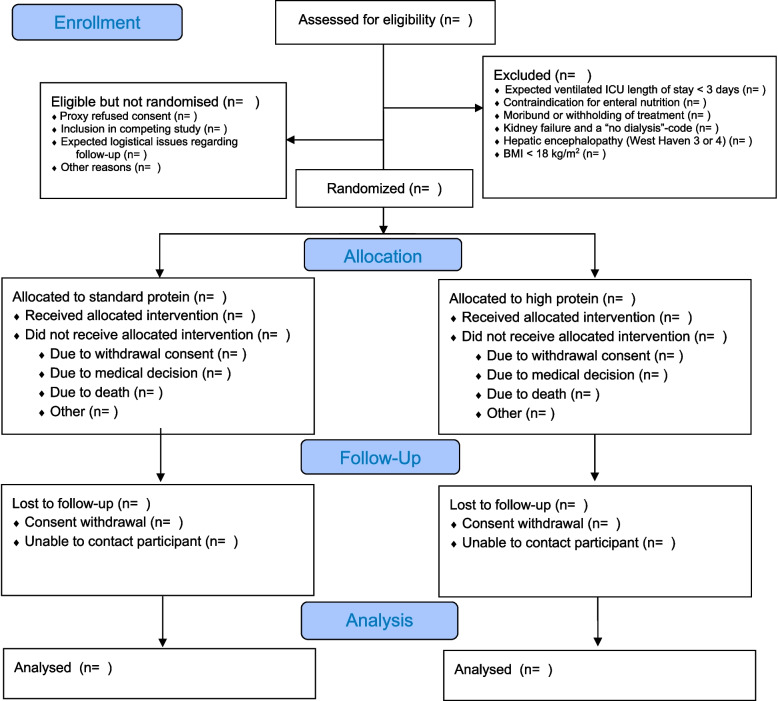
CONSORT diagram of participants in the PRECISe trial (CONSORT diagram)

A CONSORT flowchart will be reported (Fig. 1).

All analyses will be performed on an intention-to-treat basis. Additionally, a per-protocol analysis will be performed in patients in whom the allocated protocol was strictly adhered to. This is defined as patients in whom enteral nutrition was initiated within 48 h of ICU admission and continued for 72 h or more. Furthermore, overall actual provision of study nutrition must have been > 80% of prescribed during mechanical ventilation.

To assess compliance with the study protocol, the amounts of EN provided will be presented for the two study groups both as mean and median total values and as percentage of target (i.e., feeding adequacy), for calories and protein separately. Values will be presented per day for the first 10 days as well as a summary value for the entire study period (total number of days on study nutrition and percentage of total study target provided).

For the primary and secondary outcomes collected during follow-up (i.e., 30, 90, and 180 days), longitudinal data analyses will be performed using linear mixed-effects models with a 3-level structure, i.e., repeated measurements are clustered within participants and participants are clustered within centers. Therefore, the models will include a fixed effect for treatment and random effects for both center and participants [49]. A model with treatment group as an independent variable will be used to calculate overall between-group differences. A time-by-treatment interaction will be added to estimate between-group differences for each time point during the follow-up period [50]. Linear mixed-effects regression accounts for the dependency of repeated measurements within a participant, as well as for missing data resulting from the fact that not all participants may be assessed at each time point. The covariance structure between random effects and the correlation structure of longitudinal measurements will be determined by the model with the lowest Akaike information criterion (AIC).

Effect sizes with 95% confidence intervals will be reported, together with a 2-sided *P*-value. The alpha used for testing will be set at < 0.05 in all cases except for testing for interactions. In that case, an alpha of 0.10 will be used. Transformation may be used when model assumptions, such as normality, are violated.

Primary outcome will be presented as between-group differences, adjusted for baseline EQ-5D-5L. As a sensitivity analysis, we will additionally adjust the primary outcome for sex, APACHE II score, APACHE IV admission diagnosis, and NRS-2002.

Finally, we will assess the association between the total amount of calories delivered (including medication-related calories), the total amount of protein delivered, and the difference between total targeted-study feeding minus total delivered-study feeding and the primary outcome.

#### Primary outcome analysis

The treatment effect for the *primary outcome* (i.e., overall between-group difference in EQ-5D-5L health utility score) will be analyzed using a linear mixed-effects model for longitudinal data analysis following the general rules as described above. Intervention effects will be presented as an overall estimate over the 6-month period, which refers to the overall EQ-5D-5L health utility score within each group and between groups including all time points [51]. In addition, between-group differences at each follow-up moment (i.e., 30, 90 and 180 days) will be presented. The primary outcome will be presented, adjusted for baseline EQ-5D-5L health utility score.

As the EQ-5D-5L health utility score incorporates death into its score, deceased patients will not lead to missing data on the primary endpoint. Only loss to follow-up at all time points or withdrawal of consent will lead to missing data. The missing data mechanism assumed for the primary endpoint is missing at random, given the amount of covariates that reflect other health outcomes, and the proposed mixed model for longitudinal data analysis is robust with regard to this missing data mechanism.

#### Secondary outcome analysis

Regarding the main *secondary endpoints*, the treatment effects for functional secondary endpoints (SF-36, HADS, revised Impact of Events scale, EQ-5D-5L (EQ-VAS and pain question), 6-min walk test, MRC-SUM, and handgrip strength) are also assessed using linear mixed-effects models. The calculation of between-group differences, the 3-level model structure (with fixed treatment effect, random center effect and random effect for participants), reporting of effect size, and the adjustment for potential confounders is similar as described for the analysis of the primary endpoint.

For the secondary endpoint mortality, first, survival curves for both treatment arms will be constructed using the Kaplan–Meier method. Then, a Cox proportional hazards frailty model, with a 2-level structure, i.e., participants clustered within centers, will be used to investigate a treatment effect on this secondary endpoint. The adjustment for potential confounders is similar as described for the primary endpoint analysis. Additionally, crude, unadjusted hazard ratios will be reported with a 95% confidence interval. The proportional hazard assumption will be examined using the scaled Schoenfeld residuals.

Considering the large number of variables that will be collected at baseline and during ICU stay, the assumption that the missing data are related to the observed data likely applies. The secondary endpoints can be analyzed using linear mixed-effects models, as the missing data mechanism in that case is missing at random (MAR). Variables on which MAR depend will be added to the models to make sure the assumption holds.

The statistical analysis methods for tertiary outcomes will be described in the statistical analysis plan (SAP).

### Interim analyses {21b}

Other than the interim safety analysis specified for the DSMB below, no interim analyses are planned.

### Methods for additional analyses (e.g., subgroup analyses) {20b}

For the primary and secondary endpoints, exploratory subgroup analyses will be performed on the basis of index ICU admission characteristics:

- Males versus females
- Older versus younger patients
- Obese versus non-obese patients
- Medical versus surgical admission
- Patients at nutritional risk versus low nutritional risk (assessed using NRS-2002 score)
- Frail versus non-frail patients (assessed using Rockwood Clinical Frailty Scale)
- Patients with limited comorbidity versus patients with multimorbidity (assessed using Charlson Comorbidity Index)
- Sepsis versus no sepsis (assessed using SEPSIS-III criteria)
- Higher versus lower disease severity (assessed using APACHE II score)
- Acute kidney injury (AKI) vs no AKI (assessed using Kidney Disease: Improving Global Outcomes criteria)
- Patients with or without severe multi-organ failure (assessed using SOFA score)
- Traumatic brain injury versus others
- COVID-19 patients versus non-COVID-19 patient
- Difference in muscle mass on admission (assessed using BIA, muscle ultrasound, and/or computed tomography)

As exploratory analyses of post-randomization groups, we will:

- Compare patients with prolonged ICU stay (> 1 week) vs short-stay patients
- Compare patients who underwent renal replacement therapy (RRT) vs patients that did not
- Compare patients based on urea-to-creatinine ratios over the first two weeks of admission

Greater detail regarding additional analyses will be provided in the final SAP.

### Methods in analysis to handle protocol non-adherence and any statistical methods to handle missing data {20c}

Once a patient is assigned to a study group (standard or high protein nutrition), he/she will remain in that arm and all efforts will be made to provide the optimal nutrition specified for that treatment assignment. In the unforeseen circumstance that this is clinically not feasible, the patient will remain in the assigned treatment arm for statistical analysis based on the intention-to-treat principle, as it represents a normal medical situation of success and failure of delivering the planned medical therapy.

Throughout the trial, reasons for missing data after randomization will be registered. As detailed in the section regarding statistical methods for primary and secondary outcomes, linear mixed-effects models will be used to analyze the primary and secondary endpoints. Considering the large number of variables that will be collected at baseline and during admission, the assumption that the missing data mechanisms are related to the observed data likely holds. The missing data are therefore missing at random, making it possible to use linear mixed-effects models since they have the particular advantage of handling random missing data robustly when taking the mechanism into account [49]. Hence, no multiple data imputation will be done in the primary analyses.

### Plans to give access to the full protocol, participant level-data and statistical code {31c}

The full study protocol will be made publicly available via the trial website (www.preciseclinicaltrial.com). At the end of the project, the complete dataset will be available after request and registered in an online catalogue according to the FAIR principles (Findability, Accessibility, Interoperability, and Reusability) [52]. We will allow a period of 3 years following the publication of the primary paper before issuing the dataset. Statistical codes used for the primary study analysis will be provided.

## Oversight and monitoring

### Composition of the coordinating center and trial steering committee {5d}

The Maastricht University Medical Center + (MUMC +) is the sponsor of the trial and will act as overall and national coordinating center (Dr. Marcel van de Poll, Drs. Julia Bels, Drs. Rob van Gassel).

Ziekenhuis Oost-Limburg (ZOL) in Genk is the co-sponsor of the trial and will act as the national coordinating center for Belgium (Prof. Dieter Mesotten, Drs. Katrien Tartaglia, Dr. Ingrid Meex). Together, these centers will coordinate the day-to-day management of the trial, meeting once a week at a minimum.

### Trial steering committee

Dr. M. van de Poll (chief investigator/coordinator Netherlands).

Prof. D. Mesotten (principal investigator/coordinator Belgium).

Drs. J. Bels (coordinating investigator).

Prof. A. van Zanten, Dr. B. Beishuizen, Prof. E. De Waele, Dr. V. Fraipont (local investigators).

Dr. Z. Puthucheary, Dr. A. Deane, Prof. P. Weijs (independent experts).

Dr. S. van Kuijk (trial statistician).

Dr. L. Vloet, F. Demuydt (patient representatives).

Responsibilities: The TSC oversees the overall conduct of the trial and advises the trial sponsor on matters of trial execution and management.

### Data management team

Drs. M. Dictus, Drs. K. Emonds, Drs. L. van Brussel.

Responsibilities: Construction and maintenance of the eCRF, data validation, and data monitoring.

Study monitor: Drs. E. van Erp.

Responsibilities: An independent study monitor will perform on-site visits at regular intervals to assess overall study conduct and protocol compliance, ranging from the informed consent procedure to source document verification.

### Composition of the data monitoring committee, its role and reporting structure {21a}

Members of the Data Safety Monitoring Board (DSMB): Dr. L. Bruckers (statistician), Drs. R. Smets (intensivist), Prof. Dr. D.W. de Lange (intensivist) and Dr. S. van Cromphaut (intensivist).

As stated in the PRECISe DSMB charter, following enrolment of 50% of the targeted sample size, a preplanned interim safety analysis will be performed by this independent DSMB. During this closed meeting, the DSMB will receive accumulating information relating to recruitment, data quality, and missing data and protocol compliance, as well as safety data on ICU and in-hospital mortality, all blinded to treatment allocation. The safety data analyzed will be ICU and hospital mortality across the two groups. Following the interim analysis, the DSMB will provide an advice to the study sponsor (i.e., continue as planned, early discontinuation due to clear harm or proposing a protocol change) and reserve the right to make additional recommendations in their report regarding the further execution of the trial. The sponsor will have final responsibility of decision. If the sponsor does not implement the advice of the DSMB, the sponsor will send the advice and a motivation for deviation from the advice to the reviewing ethical committee. The DSMB charter can be found in the supplemental material.

### Adverse event reporting and harms {22}

Due to the nature of the patient population (i.e., critically ill patients), all participants will enter the study in a state of life-threatening illness and are likely to experience many events that could be classified as an (S)AE. This is part of the normal disease course of ICU patients and not related to participation in the study. In addition, both study interventions (either high or standard protein formulas) are part of current routine care within the ICU and known to be safe. Therefore, only the SAEs which result in death or life-threatening situations due to complications with study nutrition will be reported.

The principal investigator or the qualified person to whom this task has been delegated should assess causal relationship between an event of interest and the study nutrition on the basis of his/her clinical judgment. The causality assessment must be made based on the available information and can be updated as new information becomes available.

Although no effect of the intervention on survival is anticipated, ICU and in-hospital mortality will be assessed between both interventions as safety endpoints and are part of the interim safety analysis performed by the independent DSMB.

After completion of the trial, several events of special interest will be compared between both groups to compare the safety and harm of the two study feeds. These include:

- Incidence of ICU acquired infections (i.e., number of patients who contracted an ICU-acquired infection)
- Incidence of acute kidney injury (i.e., number of patients with Acute Kidney Injury (AKI), defined as a serum creatinine level higher than 2 times baseline level or requirement of renal replacement therapy)
- Refeeding hypophosphatemia (defined as phosphate concentration below < 0.65 mmol/l, a drop > 0.16 mmol/L from previous concentration in ICU and no other explanation for hypophosphatemia)
- Incidence of hepatic dysfunction (i.e., number of patients with hepatic dysfunction, defined as a total bilirubin level > 3 mg/dL)
- Incidence of gastrointestinal intolerance/symptoms (i.e., number of patients that experienced, at any time during index ICU stay, vomiting, ischemia, diarrhea, abdominal distention, gastric paresis, or bleeding/ulcer.)

### Frequency and plans for auditing trial conduct {23}

An independent auditor from the funders or sponsor can audit the trial conduct at any time and at any study site. All parts of the trial procedures from informed consent process to source documentation and protocol compliance can be audited.

### Plans for communicating important protocol amendments to relevant parties (e.g., trial participants, ethical committees) {25}

If, for any reason, a substantial amendment to the study protocol is necessary, an amended protocol will have to be re-evaluated by the reviewing ethical committees. Substantial protocol amendments will be communicated to all relevant parties, including investigators, trial participants, trial registries, and all committees and instances involved in trial oversight. If a substantial amendment is made, this will be adopted in the final report and manuscript of the study, which will be offered to a peer-reviewed journal for publication.

### Dissemination plans {31a}

Regardless of the outcome of the trial, a trial manuscript will be offered for publication in a peer-reviewed journal. In addition, trial results will be communicated via symposia and relevant conferences on ICU and clinical nutrition. A trial summary aimed for the general public will be produced for the trial website, communicated to interested trial participants, and shared on the funder’s website. The patient organization involved in the trial will aid in the dissemination of study results to patients.

## Discussion

The most challenging elements in the execution of the trial will be adherence to the nutritional protocol and collection of all endpoints (both questionnaires and physical tests) during follow-up. Regarding the former, all study sites will be prepared before start of the trial to perform some necessary adjustments to their daily routine to accommodate the nutritional protocol. Changes include adjustments to ordering sets for nutrition in the patient file, automated instructions for nurses to record the daily amount of nutrition given to PRECISe participants, and adjustments to local protocol concerning assessment of gastric residues and catch-up feeding. Regarding follow-up, sites will be motivated to perform the follow-up visit as completely as possible. This entails performing house visits and working together with treating physical therapists to collect all physical endpoints if a participant is unable to return to the hospital, as well as face-to-face or telephone interviews to gather all questionnaires. The PRECISe study team as well as the PRECISe trial steering committee will regularly monitor the protocol adherence, on multiple domains, and correct where necessary.

## Trial status

The study protocol for the Belgian sites (V2.0 d.d. 14–08-2020) was approved by the Belgian leading ethics committee on 2 September 2020. The study protocol for the Dutch sites (V3.0 d.d. 25–09-2020) was approved by the Dutch ethics committee on 5 October 2020. The first participant was included on 19 November 2020. After the preplanned safety interim analysis, which revealed no safety issues, the study protocol was amended only to increase sample size. Final protocol for the Belgian sites (V3.0 d.d. 15–12-2022) was approved by the Belgian leading ethics committee on 21 December 2022. Final protocol for the Dutch sites (V4.0 d.d. 15–12-2022) was approved by the Dutch ethics committee on 13 January 2023. The final participant was included in April 2023, with the last participant ‘s last visit planned for October 2023.

## Supplementary Information

**Additional file 1.**

## Data Availability

The final trial dataset will be accessible to the sponsor and study team members. The sponsor also holds authorization over contractual agreements that limit such access for investigators. Data will be made available upon reasonable request. Each request will be considered by the PRECISe TSC.

## Abbreviations

AIC: Akaike Information Criteria
AKI: Acute kidney injury
APACHE II: Acute Physiology and Chronic Health Evaluation II
ASPEN: American Society for Parenteral and Enteral Nutrition
azM/MUMC: Academisch ziekenhuis Maastricht/Maastricht Universitair Medisch Centrum
BeNeFIT: Belgium-Netherlands Funding of International Trials
BIA: Bioelectrical impedance analysis
BMI: Body mass index
CCI: Charlson Comorbidity Index
COS: Core outcome set
COVID-19: Coronavirus disease 2019
DSMB: Data Safety Monitoring Board
ECMO: Extracorporeal membrane oxygenation
eCRF: Electronic case report form
EN: Enteral nutrition
EQ-5D-5L: EuroQol 5-dimension-5L questionnaire
EQ-VAS: EuroQol visual analogue scale
ESPEN: European Society for Clinical Nutrition and Metabolism
FAIR: Findability, accessibility, interoperability, and reusability
FPI: First patient in
GCP: Good Clinical Practice
HADS: Hospital Anxiety and Depression Scale
HRQL: Health-related quality of life
ICU: Intensive care unit
IES-R: Impact of Event Scale revised
IWRS: Interactive web response system
KCE: Belgian Health Care Knowledge Centre
LPLV: Last patient last visit
MAR: Missing at random
MCS: Mental component sub score (of SF-36)
METC: Medical research ethics committee (MREC); in Dutch: medisch-ethische toetsingscommissie (METC)
MRC-SUM: Medical Research Council-SUM scale
NRS-2002: Nutrition Risk Screening 2002
OACIS: Outcomes After Critical Illness and Surgery
PCS: Physical component sub score (of SF-36)
PRECISe: PRotEin provision in Critical IllneSs
RCT: Randomized clinical trial
RRT: Renal replacement therapy
SAE: Serious adverse event
SAP: Statistical analysis plan
SD: Standard deviation
SF-36: Short Form 36 Health survey questionnaire
SOFA: Sequential Organ Failure Assessment
TSC: Trial steering committee
VAP: Ventilator-associated pneumonia
ZOL: Ziekenhuis Oost-Limburg
ZonMW: Netherlands Organisation for Health Research and Development
