# Supplementary material for: The impact of high versus standard enteral protein provision on functional recovery following intensive care admission (PRECISE trial): study protocol for a randomized controlled, quadruple blinded, multicenter, parallel group trial in mechanically ventilated patients
Source: Trials. 2023 Jun 19;24:416. doi: 10.1186/s13063-023-07380-3 (PMC10280915; doi:10.1186/s13063-023-07380-3)
Supplement: Supplementary file 1 — Additional file 1. [file 13063_2023_7380_MOESM1_ESM.pdf]

The impact of **high** versus **standard protein** provision on **functional recovery** following ICU admission

<u>Inclusion</u>

- ≥18 years
- Unplanned ICU admission
- Invasive mechanical ventilation <24 hours after ICU admission
- Expected ICU stay on mechanical ventilation of 3 days or more

<u>Exclusion</u>

- Contraindication to enteral nutrition
- Moribund or withholding of treatment
- Kidney failure AND a ”no dialysis”-code
- Hepatic encephalopathy (West Haven grade 3-4)
- BMI <18 kg/m^2

Randomization

High protein: 2.0 g/kg/day

Standard protein: 1.3 g/kg/day

End of intervention: enteral nutrition not longer required OR ICU discharge OR day 90 of ICU admission

Follow-up

30 days	90 days	180 days

Primary outcome: **EQ-5D-5L Health Utility Score** over 180 days

<u>Secondary outcomes</u>†

- Overall survival
- Short Form Health Survey (SF-36)
- Hospital Anxiety and Depression Scale (HADS)
- Impact of Event Scale - Revised (IES-R)
- 6-minute walk test
- Hand grip strength
- Medical Research Council (MRC)-sum score
- Rockwood Clinical Frailty Scale

† Needham DM et al. Core Outcome Measures for Clinical Research in Acute Respiratory Failure Survivors. An International Modified Delphi Consensus Study. Am J Respir Crit Care Med. 2017.
